# Host-Microbe Coevolution: Applying Evidence from Model Systems to Complex Marine Invertebrate Holobionts

**DOI:** 10.1128/mBio.02241-18

**Published:** 2019-02-05

**Authors:** Paul A. O’Brien, Nicole S. Webster, David J. Miller, David G. Bourne

**Affiliations:** a College of Science and Engineering, James Cook University, Townsville, QLD, Australia; b Australian Institute of Marine Science, Townsville, QLD, Australia; c AIMS@JCU, Townsville, QLD, Australia; d Australian Centre for Ecogenomics, University of Queensland, Brisbane, QLD, Australia; e ARC Centre of Excellence for Coral Reef Studies, James Cook University, Townsville, QLD, Australia; f Centre for Tropical Bioinformatics and Molecular Biology, James Cook University, Townsville, QLD, Australia; University of Texas Health Science Center at Houston

**Keywords:** codivergence, coevolution, marine invertebrates, microbiome, phylosymbiosis

## Abstract

Marine invertebrates often host diverse microbial communities, making it difficult to identify important symbionts and to understand how these communities are structured. This complexity has also made it challenging to assign microbial functions and to unravel the myriad of interactions among the microbiota. Here we propose to address these issues by applying evidence from model systems of host-microbe coevolution to complex marine invertebrate microbiomes. Coevolution is the reciprocal adaptation of one lineage in response to another and can occur through the interaction of a host and its beneficial symbiont. A classic indicator of coevolution is codivergence of host and microbe, and evidence of this is found in both corals and sponges. Metabolic collaboration between host and microbe is often linked to codivergence and appears likely in complex holobionts, where microbial symbionts can interact with host cells through production and degradation of metabolic compounds. Neutral models are also useful to distinguish selected microbes against a background population consisting predominately of random associates. Enhanced understanding of the interactions between marine invertebrates and their microbial communities is urgently required as coral reefs face unprecedented local and global pressures and as active restoration approaches, including manipulation of the microbiome, are proposed to improve the health and tolerance of reef species. On the basis of a detailed review of the literature, we propose three research criteria for examining coevolution in marine invertebrates: (i) identifying stochastic and deterministic components of the microbiome, (ii) assessing codivergence of host and microbe, and (iii) confirming the intimate association based on shared metabolic function.

## Abstract (precis)

Marine invertebrates often host diverse microbial communities, making it difficult to identify important symbionts and to understand how these communities are structured. This complexity has also made it challenging to assign microbial functions and to unravel the myriad of interactions among the microbiota.

## INTRODUCTION

Coevolution theory dates back to the 19th century (box 1), and coevolution is currently referred to as the reciprocal evolution of one lineage in response to another (1). This definition encompasses a broad range of interactions such as predator-prey, host-symbiont, and host-parasite interactions or interactions among the members of a community of organisms such as a host and its associated microbiome (1, 2). In the case of host-microbe associations, this has produced some of the most remarkable evolutionary outcomes that have shaped life on Earth, such as the eukaryotic cell, multicellularity, and the development of organ systems (3, 4). It is now recognized that microbial associations with a multicellular host represent the rule rather than the exception (4), but in complex associations of that kind, the extent to which coevolution operates is often unclear.

BOX 1: A BRIEF HISTORY OF COEVOLUTION

Charles Darwin once explained the sudden and rapid diversification of flowering plants as an “abominable mystery,” since it could not be explained by traditional views of evolution alone (5). While his correspondent Gaston de Saporta speculated that a biological interaction between flowering plants and insects might be the cause of the phenomenon, it was not until nearly 100 years later that the concept of coevolution developed. In a pioneering study, Ehrlich and Raven (6) observed that related groups of butterflies were feeding on related groups of plants and speculated this was due to a process for which they coined the name “coevolution.” Using butterflies, they argued that plants had evolved mechanisms to overcome predation from herbivores, which in turn had evolved new ways to prey on plants. Decades on, the introduction of phylogenetics has shown that plants evolved in the absence of butterflies, which colonized the diverse group of plants after their chemical defenses were already in place (7). Nevertheless, the theory of coevolution was endorsed, and two important points came to light. First, care must be taken when inferring coevolution from seemingly parallel lines of evolution, and where possible, divergence times and common ancestry should be included. Second, coevolution can occur between communities of organisms (“guild” coevolution), as observed in the case of flowering plants, where predation and pollination from a wide variety of insects likely influenced the diversification of angiosperms (8).

Since coevolution can occur across multiple levels of interactions, multiple theories have also developed. The Red Queen theory is based on the concept of antagonistic coevolution and assumes that an adaptation that increases the fitness of one species will come at the cost to the fitness of another (9). This type of coevolution has been most pronounced in host-parasite interactions, where the antagonistic interactions are closely coupled (10). However, coevolutionary patterns may also arise in the case of mutualistic symbioses, which require reciprocal adaptations to the benefit of each partner (11). Mutualistic coevolution is associated with a number of key traits that are discussed further in this review, such as obligate symbiosis, vertical inheritance, and metabolic collaboration. Third, coevolution has also recently been placed in context of the hologenome theory (12), which suggests that the holobiont can act as a unit of selection (but not necessarily as the primary unit) since the combined genomes influence the host phenotype on which selection may operate (13, 14). However, hologenome theory also acknowledges that selection acts on each component of the holobiont individually as well as in combination with other components (including the host). Thus, the entity that is the hologenome may be formed, in part, through coevolution of interacting holobiont compartments, in addition to neutral processes (12).

Given the ubiquitous nature of host-microbe associations and the huge metabolic potential that microorganisms represent, it is not surprising that evidence of host-microbe coevolution is emerging. Model representatives of both simple and complex associations are being used to study coevolution, allowing researchers to look for specific traits, signals, and patterns (1, 15). A well-known model system is the pea-aphid and its endosymbiotic bacteria in the genus *Buchnera*. This insect has evolved specialized cells known as bacteriocytes to host its endosymbionts, which in turn synthesize and translocate amino acids that are missing from the diet of the pea aphids (16). Amino acid synthesis occurs through intimate cooperation between host and symbiont, with some pathways missing from the host and some from the symbiont, such that the relationship is obligate to the extent that the one organism cannot survive without the other (17). The human gut microbiome has been extensively studied in complex systems and has been shown to be intimately associated with human health. Gut microbes have been shown to be linked with human behavior and development through metabolic processes, such as microbial regulation of the essential amino acid tryptophan (18, 19). The human microbiome contains around 150-fold more nonredundant genes than the human genome (20), and the metabolic capacity of microbes residing in the intestine is believed to have been a driving evolutionary force in the host-microbe coevolution of humans (2). In these examples, as well as many others (21–23), both host and symbiont evolved to maintain and facilitate the symbiosis. Furthermore, phylogenies of host and symbiont in these systems are often mirrored, indicating that host and symbiont are diverging in parallel (16, 24, 25), a phenomenon known as codivergence (26).

**FIG 1:**
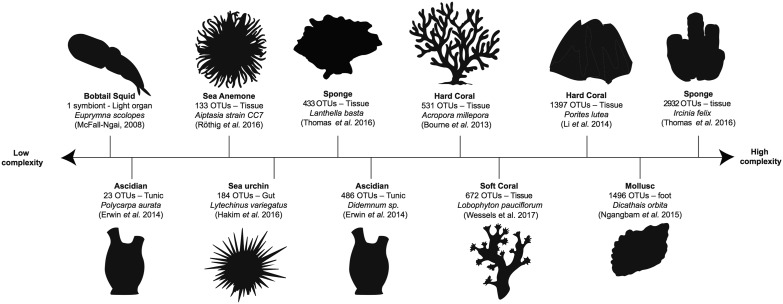
Spectrum of microbial diversity associated with different compartments of marine invertebrates. Microbial associations may involve a single symbiont in a specialized organ or over 1,000 operational taxonomic units (OTUs) associated with tissues. The levels of OTUs reported in the figure represent the highest recorded in the referenced study for that species. Reported levels of diversity may differ significantly within the same species across different studies.

In the marine environment, invertebrates can host microbial communities as simple and stable as that of the pea aphid or as complex and dynamic as that of the human gut (Fig. 1). The Hawaiian bobtail squid, for example, maintains an exclusive symbiosis with a single bacterial symbiont which it hosts within a specialized light organ (27). On the other hand, corals host enormously diverse microbial communities, comprising thousands of species-level operational taxonomic units (OTUs), which are often influenced by season, location, host health, and host genotype (28–31). Marine sponges also host complex microbial communities with diversity comparable to that of corals (32) but with associations that are generally far more stable in space and time (33). Less-diverse microbial communities are found in the sea anemone *Aiptasia*, where the number of OTUs is generally in the low hundreds (34). Due to the close taxonomic relationship of *Aiptasia* with coral and its comparatively simple microbial community, it has been proposed as a model organism for studying coral microbiology and symbiosis (34). Some marine invertebrates also include species along a continuum of microbial diversities. Ascidians, for example, have been shown to host fewer than 10 (Polycarpa aurata) or close to 500 (*Didemnum* sp.) microbial OTUs within their inner tunic (35). Furthermore, species with low microbial diversity such as P. aurata can exhibit high intraspecific variation, with as few as 8% of OTUs shared among individuals of the same species (35). Taken together, the data from those studies highlight the vast spectrum of associations that marine invertebrates form with microbial communities in terms of diversity, composition, and stability (Fig. 1).

**FIG 2:**
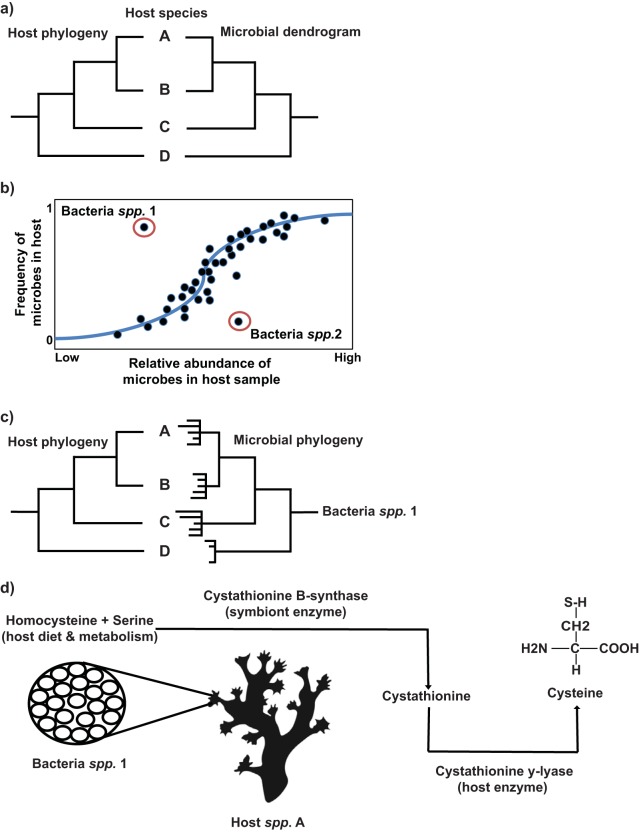
Hypothetical scenario addressing three criteria for host-microbe coevolution in species A to D. (a) Phylosymbiosis shown through hierarchical clustering of the microbial community, resulting in a microbial dendrogram which mirrors host phylogeny. (b) Neutral model showing the expected occurrence of microbes based on neutral population dynamics (blue line). As the relative abundance increases, so too does the occurrence in host samples. The members of bacterial species group 1 (Bacteria spp. 1) are therefore more abundant than would be expected by chance and may indicate active selection, while the members of Bacteria spp. 2 are less abundant. (c) Codivergence of the members of Bacteria spp. 1 with their hosts. The members of Bacteria spp. 1 are found within the microbial community of each host species and appear to be actively selected for. Their phylogeny indicates a host split at the strain level followed by diversification within each host species. Congruence between host and microbial lineages suggests important host-microbe interactions and warrants further investigation. (d) Metabolic collaboration between the members of Host spp. A and those of Bacteria spp. 1. Fluorescence *in-situ* hybridization (FISH) confirms that the members of Bacteria spp. 1 are located within bacteriocyte cells in the tissues of Host spp. A. Genome and transcriptome data for each species suggest that the amino acid cysteine is produced by the activity of a metabolic pathway shared between host and microbe. In corals of the genus *Acropora*, for example, the genome is incomplete with respect to biosynthesis of cysteine and represents a potential pathway for collaborations of host and microbe (101). Hypothetically, the amino acids homocysteine and serine (potentially sourced from host diet and metabolism) are combined to form cystathionine through the enzyme cystathionine V synthase (provided by the host’s endosymbiont). The host enzyme cystathionine γ-lyase then breaks down cystathionine to form cysteine.

While previous research has provided a good understanding of the composition of marine invertebrate microbiomes, our understanding of how the microbiome interacts with the host, and of the potential to coevolve, is far more limited. Moreover, the increasing number of studies generating tremendous volumes of host-associated microbiome sequence data requires theoretical development to interpret these relationships. Coevolved microbial symbionts are presumed to be intimately linked with host fitness and metabolism (36); therefore, understanding these relationships in marine invertebrates will have direct implications for health and disease processes in these animals. Three research criteria arise for examining coevolution in marine invertebrates: (i) identifying stochastic and deterministic microbial components of the microbiome, (ii) assessing codivergence of host and microbe, and (iii) confirming an intimate association between host and microbe related to shared metabolic function (metabolic collaboration). While each of these criteria may be fulfilled without the involvement of coevolution (26, 37, 38), evidence of their existence in combination provides a strong basis for establishing coevolution patterns (Fig. 2). This review positions these three criteria in coevolution as representing a complementary approach to the study of complex marine invertebrate microbiomes by drawing from examples of model systems. Focussing on keystone coral reef invertebrates, this review also evaluates the current evidence for each criterion. Finally, while parasites and pathogens also contribute to host coevolution, the focus of this review is mutualistic symbionts; thus, pathogens and parasitism are not discussed.

BOX 2: GLOSSARY

**(i) Codivergence.** Two organisms which speciate or diverge in parallel as illustrated by topological congruency of phylogenetic trees.

**(ii) Coevolution.** Reciprocal adaptation of one (or more) lineage(s) in response to another (or others).

**(iii) Holobiont.** A host organism and its associated microbial community.

**(iv) Hologenome.** The collective genomes of a host and its associated microbial community, which may act as a unit of selection or at discrete levels.

**(v) Metabolic collaboration.** Two or more oganisms that are linked through metabolic interactions, generally to the benefit of one another.

**(vi) Metagenome.** The collective microbial genes recovered from an environmental sample, usually predominantly prokaryotic.

**(vii) Metatranscriptomics.** Quantification of the total microbial mRNA in a sample as an indication of gene expression and active microbial functions.

**(viii) Microbiome.** The total genetic make-up of a microbial community associated with a habitat.

**(ix) Microbiota.** The community of microorganisms residing in a particular habitat, usually a host organism.

**(x) Phylosymbiosis.** The rentention of a host phylogenetic signal within its associated microbial community.

**(xi) Virome.** The total viral genetic content recovered from an environmental sample.

## UNTANGLING PATTERNS OF HOST-MICROBE COEVOLUTION IN A WEB OF MICROBES

### (i) Phylosymbiosis and neutral theory—identifying stochastic and deterministic components of the microbiome.

Host-microbe coevolution may occur to some degree at the level of the hologenome, i.e., reciprocal evolution of the host genome and microbiome (12). Therefore, it is necessary to understand microbial community structure and population dynamics within the host environment. This may illustrate (i) that the microbiome associated with a host is structured through phylogenetically related host traits and may therefore retain a host phylogenetic signal (phylosymbiosis) and (ii) that certain microbes deviate from the expected patterns of neutral population dynamics, i.e., stochastic births and deaths and immigration. It is likely that phylosymbiosis and neutral population dynamics are linked; therefore, their potential to contribute to coevolution is discussed together.

The term “phylosymbiosis” is not intended to imply coevolution (12, 38); however, coevolution of a host and microbiome may reinforce patterns of phylosymbiosis. There are many host traits that correlate with host phylogeny, some of which can act as environmental filters, preventing the establishment of microbes in the host environment. Thus, neutral population dynamics, with host traits acting as an ecological filter to microbial immigration, may be sufficient to result in phylosymbiotic patterns (39, 40). However, host traits are not static; thus, the evolution of these microbial niches may further drive the radiation of the microbes that reside within them. In turn, the continuous colonization over many generations of a microbial community likely adds to the selective pressure on host traits. Therefore, ecological filtering of microbes through host traits and coevolution of a host and microbiome need not be mutually exclusive in the appearance of phylosymbiosis (39). Moreover, assessing patterns of phylosymbiosis and neutral population dynamics also allows the detection of microbes that deviate from these patterns and may identify important microbial species that are actively selected for (or against) by the host. In this context, neutral models can simulate expected microbial abundance, allowing easier detection of microbes that do not fit these patterns (41). This reasoning justifies consideration of phylosymbiosis and microbial population dynamics in assessing coevolution in complex holobionts.

Patterns of phylosymbiosis are frequently detected in complex holobionts. One particular study tested for phylosymbiosis across 24 species of terrestrial animals from 4 groups that included *Peromyscus* deer mice, *Drosophila* flies, mosquitos, and *Nasonia* wasps and an additional data set of 7 hominid species (42). Since these animals (with the exception of hominids) could be reared under controlled laboratory conditions, environmental influences could be eliminated, leaving the host as the sole factor influencing the microbial community. Under these conditions, phylosymbiotic patterns were clearly observed for all five groups, with phylogenetically related taxa sharing similar microbial communities and microbial dendrograms mirroring host phylogenies. Similar patterns of phylosymbiosis have been observed in a growing number of terrestrial systems, including all five gut regions in rodents (43), the skin of ungulates (44), the distal gut in hominids (45), and roots of multiple plant phyla (46), providing evidence that such patterns are common among host-associated microbiomes.

In the marine environment, two major studies, one involving 236 colonies across 32 genera of scleractinian coral collected from the east and west coasts of Australia (47) and the other involving 804 samples of 81 sponge species collected from the Atlantic Ocean, Pacific Ocean, and Indian Ocean and the Mediterranean Sea and Red Sea (32), have provided the most convincing examples of phylosymbiosis. Both studies found a significant evolutionary signal of the host with respect to microbial diversity and composition. Specifically, mantel tests were used to delineate the finding that closely related corals and sponges hosted more extensively similar microbial communities in terms of composition than would be expected by chance. In the case of corals, the similarity was seen in the skeleton and, to a lesser extent, in the tissue microbiome, while the mucus microbiome was more highly influenced by the surrounding environment (47). However, both studies found that host species was the strongest factor in explaining dissimilarity among microbial communities. Additional studies on both cold water and tropical sponges have found similar phylogenetic patterns within the microbiome of the host species (48, 49). Together, these results suggest that host phylogeny (or associated traits) has a significant role in structuring associated microbial communities, although there are additional factors related to host identity (and unrelated to phylogeny) that also likely play a major role.

Most studies to date have focused on the microbes that adhere to these patterns of phylosymbiosis, though more-useful information arguably could be determined from the microbes that do not. Since phylosymbiosis is a pattern that shows correlations between microbiome dissimilarity and host phylogeny, it does not indicate active microbial selection or cospeciation (38), and the species that deviate from these patterns would be interesting targets for studies of codivergence and metabolic collaboration (see below). Neutral models have been applied to three species of sponges, a jellyfish, and a sea anemone, and while neutral models have been shown to fit well to the expectation of microbial abundance in sponges (which also show phylosymbiosis), jellyfish and sea anemone microbiomes were found to be associated with a higher level of nonneutrality (40). Potential reasons for nonneutrality include the presence of a more sophisticated immune system in cnidarians that provides active selection on certain microbial taxa and that the microbiomes in such cases are more transient or a combination of the two. In summary, neutral population dynamics filtered through phylogenetically related host traits likely result in, or at least contribute to, the observed patterns of phylosymbiosis. This does not necessarily mean that the pattern is unimportant or is not contributing to coevolution at the hologenome level, and it may be that the communities of microbes that follow these patterns are responsible for broad ecological functions (50). On the other hand, microbes that deviate from these patterns may be responsible for more-specific functions and are of high interest to those trying to identify symbionts and coevolution at the microbial species or strain level.

### (ii) Codivergence—microbial phylogeny and host phylogeny are congruent.

The second criterion in assessing host-microbe coevolution is that of whether individual microbial lineages and their hosts have matching phylogenies (22, 24, 51). Codivergence implies a tightly coupled, long-term interaction between two species and can potentially identify beneficial symbionts (or parasites) that have coevolved with the host (26). However, it is also important that codivergence can arise due to processes other than coevolution, such as one species adaptively tracking another, which would imply that the evolution is not reciprocal, or two species responding independently to the same speciation event or environmental stress (37). In known cases of coevolution, phylogenies of hosts and their microbial symbionts are congruent (16, 51, 52). However, in complex and uncharacterized systems, this strategy can be reversed to identify potential symbionts. Therefore, the main value of investigating codivergence in complex associations is to identify those specific microbes on which to focus further attention.

Codivergence has been demonstrated in the case of Hydra viridissima, a freshwater relative of marine cnidarians, and its photosymbiont *Chlorella* (53). In this system, photosynthetically fixed carbohydrates from *Chlorella* are transported to its host (54), and phylogenetic analysis of 6 strains of H. viridissima and their vertically transmitted symbionts revealed clear congruency of host and symbiont topologies (55). In more-complex systems, patterns of codivergence have been illustrated in the gut microbiota of hominids (25). Analysis of fecal samples from humans, wild chimpanzees, wild bonobos, and wild gorillas showed that four clades of bacteria from the dominant families *Bacteroidaceae* and *Bifidobacteriaceae* codiverged with host phylogeny. Importantly, this example illustrates one possible way of identifying codivergence in complex holobionts where the symbionts are unknown. Since bacteria from the families *Bacteroidaceae*, *Bifidobacteriaceae*, and *Lachnospiraceae* are known to dominate the gut of hominids, multiple primer sets targeting each individual family were utilized, and phylogenetic analyses of the families were completed independently. Furthermore, instead of using the relatively slowly diverging 16S rRNA gene, the fast-evolving and variable gene encoding DNA gyrase subunit B was used for bacterial phylogenetics. Similar methods may be applied to complex marine invertebrates such as coral and sponges, where 16S rRNA gene studies have identified prominent bacteria.

Within complex marine invertebrate holobionts, codivergence has been most clearly demonstrated in cold-water sponges in the family Latrunculiidae. The microbiomes of six species within this family were dominated by a single betaproteobacterial OTU, and the phylogeny of this OTU was highly congruent with that of the host (56). Furthermore, gene expression analysis suggested that the dominant betaproteobacteria are active members of the microbiome rather than dormant or nonviable members; however, whether or not this potential symbiont and its host participate in metabolic collaboration is unknown, highlighting an example warranting further investigation. The microbiomes of many other marine invertebrates are dominated by members of the genus *Endozoicomonas* (57). A pan-genomic analysis of the genomes of seven *Endozoicomonas* strains representing a broad range of hosts (corals, sponges, and sea slugs) provided some evidence for codivergence (58). Strikingly, the two closely related corals Stylophora pistillata and Pocillopora verrucosa hosted *Endozoicomonas* with highly similar genomes. A second, large-scale study (47) found that *Endozoicomonas* species within the coral tissues showed strong signals of codivergence with their hosts; however, they were grouped into two major divisions, namely, the host-specific and host-generalist divisions. The presence of a host-generalist clade may partly explain why the patterns of codivergence did not hold when samples of S. pistillata and P. verrucosa were collected across 28 reefs worldwide (59). Furthermore, the genome of *Endozoicomonas* is large and appears to be adapted to a planktonic lifestyle (57). Having a free-living stage with respect to the *Endozoicomonas* life cycle suggests a facultative relationship with corals and would limit the extent of codivergence.

Codivergence may also occur between two symbionts within the microbial community associated with a single host. An interesting example occurs in lower termites, which live in a symbiotic relationship with flagellate protozoa that are essential for the breakdown of lignocellulose obtained from wood particles (60). Within the hindgut, these flagellate protozoa are associated with endosymbiotic prokaryotes, and while the functional basis of this relationship is unclear, matching phylogenies of flagellate host and prokaryote symbiont indicate codivergence (61). The microbiomes of many marine invertebrates also include both eukaryotes and prokaryotes that appear to closely interact with one another. For example, the symbiotic algae Symbiodiniaceae, which reside in the endoderm of the coral tissue, are producers of dimethylsulfoniopropionate (DMSP), which is thought to be metabolized by bacteria within the holobiont (62). Symbiodiniaceae and bacteria are also linked through the nitrogen cycle, where diazotrophs within the holobiont are postulated to fix nitrogen such that it can be used by the endosymbiotic algae (63, 64). Furthermore, the existence of a core microbiome associated with Symbiodiniaceae appears likely, with bacteria affiliated to *Marinobacter*, *Labrenzia*, and *Chromatiaceae* present across 18 cultures of Symbiodiniaceae spanning 5 genera (65). A range of other marine invertebrates, including soft corals, sponges, and molluscs, also host Symbiodiniaceae, and it would be valuable to investigate whether Symbiodiniaceae show codivergence and coevolution with prokaryotes in these systems.

### (iii) Metabolic collaboration—intimate association between host and microbe.

A third key feature of coevolution is that host and microbe collaborate in a way that is mutually beneficial (15). This is often related to the metabolic function of the microbe, with the host facilitating or complementing that function. This could be in the form of a specialized cell or organ to host microbial symbionts (27), a shared metabolic pathway to produce essential vitamins or amino acids (17), or microbial regulation of certain metabolites produced by the host (19). Metabolic collaboration should be validated where potential candidates for coevolution have been identified through population dynamics and codivergence, as reciprocal evolution necessitates an interaction between the two species. A key step in demonstrating an interaction, and therefore identifying potential reciprocal evolution, is to look at the genome and transcriptomes of the host and symbionts for evidence of integrated metabolism, combined with targeted *in situ* visualization of metabolite passage to support the metabolic collaboration.

Sharpshooters, a group of xylem-feeding insects, provide an elegant example of metabolic collaboration between a host and bacterial symbionts. Sharpshooters host two microbial symbionts, Baumannia cicadellinicola and Sulcia muelleri, in their specialized bacteriocyte cells (36), and both symbionts show patterns of codivergence with their host (66). The genomes of B. cicadellinicola and S. muelleri predict the synthesis of vitamins and essential amino acids, respectively, which are deficient in the diet of sharpshooters (23). Furthermore, these two symbionts not only appear to complement each other in terms of their roles in supplementing the host diet, but each symbiont also appears dependent on the other. Circumstantial evidence suggests that similar functional relationships may exist among marine invertebrates, and the characterization of these should be a high priority.

Some examples of metabolic collaboration in complex marine invertebrate holobionts are represented by sponges. Genome and transcriptome data from *Cymbastela concentrica* and two of its bacterial symbionts (novel genomes of the *Phyllobacteriaceae* and *Nitrosopumilales*) suggest that creatine and creatinine produced by sponge metabolism are likely to be degraded to the amino acid glycine by its symbionts (67). Furthermore, gene expression data suggest that the urea produced by creatine degradation by the *Phyllobacteriaceae* symbiont may be transported and degraded by a third bacterial symbiont in the genus *Nitrospira* (67). The potential for metabolic collaboration also exists between the sponge Theonella swinhoei and its symbiont belonging to “*Candidatus* Entotheonella.” The genome of “*Ca.* Entotheonella” possesses the repertoire for production of almost all amino acids as well as rare coenzymes; however, additional research is needed to understand if these products are used by the host (68). While the following does not constitute metabolic collaboration, sponge symbionts also appear to interact with their host through eukaryote-like proteins (ELPs). For example, microbial symbionts associated with different sponges often contain genes coding for ELPs, some of which are phylogenetically similar to those found in sponges and appear to inhibit phagocytosis (69, 70). Furthermore, additional functional domains associated with ELPs suggest that these proteins are transported to the outer membrane, where they are maintained and potentially used in bacterium-host interactions (71). A symbiosis maintained through host-bacterium interactions such as this emphasizes the potential for coevolution to take place, although it does not in itself demonstrate reciprocal evolution. Finally, characterizations based on metagenomic and metatranscriptomic data sets require functional validation using techniques such as stable isotope probing (SIP) (for a review, see reference 72). For example, using ^14^C- and ^13^C-labeled bicarbonate in combination with autoradiography and nanoscale secondary ion mass spectrometry (nanoSIMS), symbionts of the colonial ciliate Zoothamnium niveum were shown to fix inorganic carbon and translocate organic carbon to its host (73). In the advent of new technology associated with SIP, future research would benefit from validating the putative microbial functions implied by genomic research.

## CORE MICROBIOME AND THE POTENTIAL OF VIRUSES

A core microbial community, i.e., one that has high intraspecies stability, is often the primary focus of microbial ecologists trying to distinguish functionally important taxa from commensals or short-term visitors (74). While a few bacterial lineages have been shown to occur across a large number of corals and other invertebrate species (57, 75), evidence of the existence of a defined and stable core community remains elusive. From a taxonomic perspective, a core community may not exist; instead, a core functional capacity may exist across diverse lineages. In marine sponges, for example, different host species associate with different symbionts that perform equivalent functions (95). Namely, host-specific microbes among different sponge species appear to use different enzymes to perform the same functions in processes such as denitrification and ammonium oxidation. However, functional redundancy in microbial ecosystems may not be as common as previously thought, as rare microbial phylotypes have been implicated in specific microbial pathways, while more-abundant phylotypes are positively correlated with broader metabolic functions such as respiration (50). This may have important implications in looking at neutral population dynamics, as those rare taxa that are present more often than expected could be responsible for key microbial functions. The existence of a core community would have obvious implications for coevolution, as universally associated microbes are more likely to have coevolved with their host. If present, reconstruction of phylogenetic relationships of core taxa can illustrate whether microbes also diverge in parallel with their host, leading to further investigations that utilize integrated genomic techniques to identify core functional genes and pathways.

While research on the microbiome of marine invertebrates has focused mostly on prokaryotes and microbial eukaryotes (box 3), there is increasing recognition of the importance of viruses as components of the holobiont, adding to the complexity of an already challenging system (76). Viruses are the most abundant biological entities in the oceans (77) and are likely to play important roles in host-microbe coevolution, as bacteria commonly acquire genes for symbiosis or pathogenicity through lateral gene transfer from viruses (78). For example, the bacterium Hamiltonella defensa is a common symbiont of aphids providing defense against wasp parasitism. However, toxin-encoding genes required for aphid protection occur only after infection from a lysogenic lambdoid bacteriophage (79). Thus, it is feasible that coevolution of host and symbiont can be made possible through the initial acquisition of symbiont genes from viruses. Furthermore, viruses structure bacterial communities through processes such as cell lysis, thereby adding another form of selective pressure to invertebrate holobionts (80). A recent study found that viral communities of corals and sponges are specific to their host species and are distinct from the viral communities inhabiting the surrounding seawater (81). Viruses of the order *Caudovirales* (tailed bacteriophages) were found across all viromes in the study, often as the dominant member; thus, a host-specific virome combined with a host-specific microbiome could be associated with viral selection and pressure. As a result, by influencing microbial community structure, viruses can have major effects on coevolution within the holobiont. The extent to which viruses influence marine invertebrate holobionts is still unknown; however, future research on reef holobionts would benefit from including analyses of both the viral and prokaryotic communities.

BOX 3: SYMBIODINIACEAE—AN OBLIGATE SYMBIONT AND A COEVOLVED PARTNER?

Dinoflagellates from the family Symbiodiniaceae (see reference 82 for revised taxonomy) are common symbionts of many different marine invertebrates, including cnidarians, sponges, molluscs, and protozoans (83). These photosynthetic dinoflagellates provide their host with fixed carbon and in return gain inorganic nutrients and a suitable living environment, creating a remarkable symbiosis that is responsible for the foundation of coral reef ecosystems (83, 84). The symbiotic lifestyle often leads to a reduction of genome size, and, although the genomes of Symbiodiniaceae are large by comparison with those of many other eukaryotic microbes, they are among the smallest for dinoflagellates. The relatively small genomes typical of the Symbiodiniaceae suggest some degree of adaptation to life inside the host (71), despite the fact that many members of this family are known to have a free-living stage (68, 69). An important exception to this life cycle is the dinoflagellate formerly known as clade C15, which is vertically transmitted in coral hosts, and culturing experiments suggested that it is unlikely that the strain can survive outside the host environment (85). Moreover, this symbiont appears to have lost its genomic potential for motility, representing a likely adaptation to life inside a host (85).

## CHALLENGES, FURTHER CONSIDERATIONS, AND CONCLUSIONS

Illustrating reciprocal adaptation of one lineage in response to another is extremely challenging in complex symbiotic systems. While meeting the basic criteria set out in this review does not prove coevolution, it would provide support for the idea of coevolution in host-microbe systems where little is known about the evolutionary origins. In doing so, it is also likely that obligate microbes can be differentiated from transient members of the holobiont. Many factors need to be considered, including common ancestry, the origins of the host-microbe association, and the estimated times of divergence. The butterfly-plant example (box 1) highlights the necessity to distinguish the possibility of microbes colonizing their host after host evolution has taken place. In the case of the aphid-*Buchnera* symbiosis, the origin of infection has been dated at 150 to 250 million years ago (MYA), when aphids first diverged from a common ancestor, and *Buchnera* form a monophyletic group that is exclusively associated with aphids (16, 36). Within hominids, divergence times were calculated for gut bacteria that show codivergence with their host and were found to coincide with host evolution. Furthermore, the hominid-microbe association appears to have arisen from a common ancestor of all African great apes.

Vertical versus horizontal microbial acquisition may also influence patterns of evolution and should be considered within any study on host-microbe coevolution. Generally speaking, microbes that are acquired vertically, i.e., passed from parent to offspring, are more likely to have coevolved with their host. This is the case for many insect endosymbionts, and their loss of a free-living stage and their subsequent adaptation to the host environment determined many of the coevolution signals previously detailed (23, 36, 86). For example, *Buchnera* endosymbionts have been passed from parent to offspring for over 100 million years and, as the endosymbiont evolved, it lost many genes required for life outside the host (16). Such patterns may be far more difficult to observe in microbes acquired from the environment (horizontal transmission). Codiversification is more difficult to detect in horizontally acquired symbionts, as the selection pressures include environmental forces that act in concert with the host-imposed pressures. Invertebrates such as cnidarians and sponges can acquire microbial symbionts through both vertical transmission and horizontal transmission (87–91), and focusing initially on vertically transmitted microbes would simplify the search for coevolutionary signals.

Consideration of genetic markers and key traits of symbiosis could also be useful for identifying potentially coevolved symbionts. For example, many vertically transmitted endosymbionts have reduced genome sizes compared to their free-living relatives, since many genes may become redundant during adaptation to the host environment (36, 86). Some microbial symbionts are also housed in bacteriocytes or other specialized compartments, and microbial aggregates resembling such associations have been detected in both corals and sponges (102, 103). Microbes housed in these specialized cells represent priority candidates in the search for coevolved relationships. Other trends, such as lower G+C content, high isoelectric point values, and proteins that are quickly evolving relative to those seen with free-living bacteria, are all features of insect endosymbionts (23). Exploring these traits in more-complex systems may also have some utility in the search for coevolved symbionts. Furthermore, observing support for host-symbiont coevolution may require careful choices of appropriate genetic markers due to different divergence rates. In particular, it has been suggested that immune genes should be targeted as they are rapidly evolving and likely to directly influence the microbial community (92). Additionally, unresolved host and microbe genealogies may further confuse patterns of host-microbe coevolution; thus, robust phylogenetic trees and markers are critical to illustrate codivergence.

To begin investigating host-microbe coevolution in complex holobionts, it may be useful to unify studies by investigating a number of model organisms. Marine sponges present an ideal starting point for investigating coevolution in complex systems for a variety of reasons. First, they may represent the earliest animal lineage to have diverged and they host highly stable microbial communities, increasing the likelihood of discovering coevolved symbionts. Second, metagenomic analyses in sponges are currently better developed than in other marine invertebrates with complex microbiomes, providing a solid platform with which to investigate coevolution. Third, some evidence of coevolution already exists, with sponges exhibiting codivergence and metabolic collaboration and some species hosting microbial cells within bacteriocytes. However, as yet, no research has traced all the aforementioned traits to a single holobiont species.

In this era of climate change and environmental degradation heavily impacting marine ecosystems (93, 94), there is an urgent need to better understand the microbial processes that underpin invertebrate health and evolution. Following the criteria set out in the review will not only enable exploration of evidence for coevolution but also provide a better understanding of how microbial communities are structured and identify potentially beneficial symbionts which can be targeted using genomic techniques to elucidate their specific roles within the holobiont.
